# Rhizodegradation of Pyrene by a Non-pathogenic *Klebsiella pneumoniae* Isolate Applied With *Tagetes erecta* L. and Changes in the Rhizobacterial Community

**DOI:** 10.3389/fmicb.2021.593023

**Published:** 2021-02-23

**Authors:** Jina Rajkumari, Yashmin Choudhury, Kasturi Bhattacharjee, Piyush Pandey

**Affiliations:** ^1^Department of Microbiology, Assam University, Silchar, India; ^2^Department of Biotechnology, Assam University, Silchar, India

**Keywords:** *Klebsiella pneumoniae*, pyrene, rhizoremediation, *Tagetes erecta* L, metagenomic analysis

## Abstract

The non-clinical *Klebsiella pneumoniae* variants, isolated from different environments, are now well acknowledged for their role in plant-growth promotion and biodegradation of pollutants. In the present study, a non-clinical environmental isolate *K. pneumoniae* AWD5 is being described for rhizoremediation of pyrene, applied through the rhizosphere of an ornamental plant, *Tagetes erecta* L (marigold). The non-pathogenic nature of AWD5 was established using an *in vivo* mouse model experiment, where AWD5 was unable to cause lung infection in tested mice. Degradation of pyrene, in the presence of succinate as co-substrate, was observed to be 87.5% by AWD5, after 21 days of incubation in minimal (Bushnell–Hass) medium *in vitro* conditions. Consequently, the bacterial inoculation through the rhizosphere of *T. erecta* L. plants resulted in 68.61% degradation of pyrene, which was significantly higher than control soil. Inoculation of AWD5 also improved plant growth and exhibited an increase in root length (14.64%), dry root weight (80.56%), shoot length (3.26%), and dry shoot weight (45.35%) after 60 days of incubation. *T. erecta* L., an ornamental plant, was also found to be suitable for bioremediation of pyrene. The effect of AWD5 application, and rhizoremediation process, on rhizosphere bacterial diversity and community structure has been studied using the metagenomic analysis of the 16S (V3–V4) region of rRNA. 37 bacterial phyla constituted the core microbiome, which was dominated by Proteobacteria followed by Actinobacteria, Actinobacteria, and Planctomycetes for all the treatments. AWD5 inoculation enhanced the relative abundance of Firmicutes and Acidobacteria as compared with other treatments. Genus *Kaistobacter* and *Verrucomicrobia* were found to be an abundant indigenous population in pyrene-spiked soils. Bacterial richness and diversity were analyzed using the Shannon–Wiener (H) index. A lower diversity index was observed in pyrene-spiked soils. Canonical correspondence analysis (CCA) showed a possible linkage with plant growth attributes and available nitrogen content that influences diversity and abundance of the bacterial community.

## Introduction

Polycyclic aromatic hydrocarbons (PAHs) are organic contaminants consisting of more than one fused aromatic ring and commonly found in a different environment such as atmosphere, freshwater, marine sediments, and soil. There has been quite an increase in the release of PAHs due to anthropogenic activities and atmospheric deposition from natural sources (Juhasz and Naidu, 2000). Exposures to these compounds showed genotoxic, mutagenic, and carcinogenic effects and are a potent immunosuppressant in human health (Rengarajan et al., 2015). Due to its widespread distribution, PAH contamination has become hazardous to the stability of soil ecosystems, and it has provoked global concern (Ren et al., 2015).

The extent of PAH biodegradation had been suggested to be dependent on the presence of benzene rings and the impact of environmental factors (Bamforth and Singleton, 2005). Pyrene is a high-molecular-weight PAH with four fused benzene rings being identified as priority pollutants by the United States Environmental Protection Agency (US EPA) (Cerniglia, 1992). Its hazardous effects comprise neurological problems, bodyweight loss, and kidney lesions (Patri et al., 2009). Pyrene serves as an ideal compound for studying the bioremediation of higher-molecular-weight PAHs as its structure resembles other carcinogenic PAHs (Peng et al., 2008). It is commonly found as contaminants in aquatic and soil environments, in coal tars, and in creosotes which are used for wood treatment (Prince and Drake, 1999). The use of microorganisms for remediation of PAHs has been regarded as the main natural degradation method in soils, as native microorganisms could utilize bioavailable PAHs as their sole source of carbon (Haritash and Kaushik, 2009). Several bacterial species (*Acinetobacter*, *Alcanivorax*, *Cellulomonas*, *Deitzia*, *Micrococcus*, *Marinobacter*, *Pseudomonas*, *Paenibacillus*, *Sphingomonas*) have been reported for degrading PAHs (Maki et al., 2010; Alegbeleye et al., 2017). *Mycobacterium* spp. have been described as efficient degraders of pyrene; besides *Rhodococcus* sp., *Novosphingobium pentaromativorans*, *Stenotrophomonas maltophilia* VUN10003, and *Leclercia adecarboxylata* PS4040 had also been identified to utilize pyrene (Klankeo et al., 2009).

Non-pathogenic *K. pneumoniae* is gradually getting attention for its non-clinical roles in the environment. The *K. pneumoniae* isolates from plants had been capable of increasing the growth of plants by associating in roots as endophytes (Riggs et al., 2001) and capable of nitrogen fixation in certain grasses (Sevilla et al., 1998; Iniguez et al., 2004). *Klebsiella* strains have been isolated from different crop plants, such as rice (An et al., 2001), sugarcane (Ando et al., 2005), maize (Chelius and Triplett, 2000), and banana (Martínez et al., 2003). *Klebsiella* spp. enhance plant growth by promoting phosphate solubilization and phytohormone production, increasing uptake of nutrition, and controlling environmental stress (Gamez et al., 2015, 2016). However, their role in the environment for biodegradation of aromatic compounds is now being realized (Rajkumari et al., 2018; Li et al., 2020).

Some researchers have raised concerns about the limitation of bioremediation, relying on microorganisms alone for PAH degradation, for the slow rate of degradation of higher-molecular-weight PAHs (Huang et al., 2004). This suggested that there is a need for improvement in the bioremediation of PAHs in soil. Some of the selected plants such as *Zea mays* L., *Trifolium repens*, *Lolium perenne* L. (Xu et al., 2006), *Apium graveolens*, *Raphanus sativus*, *Solanum tuberosum*, *Daucus carota* (Yi and Crowley, 2007), *Medicago sativa* L., *Bromus inermis* (Sheng-Dong et al., 2016), flowering plant *Helianthus annuus* L. (Tejeda-Agredano et al., 2013), and *Hippophae rhamnoides* L. (Shevchyk and Romaniuk, 2016) had been reported for bioremediation of pyrene-contaminated soil. Plant roots enhance bacterial density and increase the surface contact between microorganisms and pollutants, leading to the promotion of degradation in the rhizospheric soil (Henner et al., 1997). Previous studies showed that ryegrass root exudates induced shifts in microbial communities’ pattern in the rhizospheric soil and degraded organic pollutants (Corgie et al., 2004, 2006). These indicated that plants increase the efficiency of microbial degradation of PAHs in soil.

Phytoremediation for hydrocarbon degradation is ordinarily accomplished by the indigenous population in rhizospheric soil. The removal efficiency of hydrocarbon is related to specific bacterial communities with the selective effect of phytoremediation rather than bacterial diversity (Hou et al., 2015). The existence of plant proliferates heterogeneity of the bacterial population; rhizospheric soil has varied microbial density; and activity and structure of the microbiome depend on different plant species (Garbeva et al., 2008; Berg and Smalla, 2009). The introduction of plants and specific bacterial strains influences the structure and diversity of bacterial communities (Bell et al., 2013a).

Tejeda-Agredano et al. (2013) observed shifts in bacterial soil community structure in the sunflower rhizosphere with an increase in the relative abundances of PAH-degrading genera. Inoculation of bacterial strains capable of PAH degradation has revealed to correlate with enhancement in relative abundance at the genus level with a high removal efficiency of C8–C16 PAHs and C21–C34 PAHs (Hou et al., 2015). This study was aimed to assess the effectiveness of *K. pneumoniae* AWD5 applied through the *Tagetes erecta* L. (marigold) rhizosphere, for the bioremediation of pyrene-contaminated soil. Here, *T. erecta* L. has been used for the application of AWD5 for pyrene bioremediation, and therefore, the changes in the rhizospheric bacterial community were also analyzed using 16S rRNA metagenome analysis targeting V3–V4 regions with specific primers using Illumina chemistry.

## Materials and Methods

### Bacterial Strain and Growth Conditions

Pure culture of bacterial strain AWD5 used in this study was obtained from the Soil and Environmental Microbiology Laboratory (SEML), Department of Microbiology, Assam University, Silchar, India. The isolate AWD5 had already been identified (*Klebsiella pneumoniae*) and characterized at the genomic level (Rajkumari et al., 2017, 2018). The strain grows best at a pH range of 4–8 in a nutrient medium at 30°C (Rajkumari et al., 2018). Inoculation of this bacterial isolate had been observed to increase the root length of the rice plant and produce a good amount of indole acetic acid (IAA), a siderophore in pyrene-contaminated soil (Singha et al., 2018). This isolate was found to improve the growth of *Jatropha curcas* in PAH-contaminated soils. Genome sequencing of *K. pneumoniae* AWD5 revealed the presence of multiple genes that contribute to plant growth promotion and degradation of aromatic compounds with no virulent factors (Rajkumari et al., 2018).

### Confirmation of the Non-pathogenic Nature of the Isolate AWD5

Female LACA Swiss albino mice (age, approximately 6 to 8 weeks, weighing 25–35 g) were obtained from Pasteur Institute, Shillong, India. Before experimentation, mice were acclimatized for 2 weeks in polypropylene wire mesh cages with wood shavings as bedding under temperature (22 ± 2°C) and relative humidity of 65%–75% and given feed ingredients and water *ad libitum*. Mice were infected by an intranasal infection model as described by Saeland et al. (2000); Erlendsdottir et al. (2001), and Fouts et al. (2008) with slight modification. The bacterial suspension (10 μL) containing ∼5.5 × 10^7^ was allowed to be aspirated through the nose while mice were hooked on a string by upper incisors for 2–3 min before returning to their cages. Three mice were treated with *K. pneumoniae* AWD5, and three untreated mice were used as a control in the trials. The mice were inoculated with the same dose for 10 days and sacrificed by cervical dislocation after 3 days of observation, and lungs, spleen, and liver were removed and transferred to sterile saline and the final volume was made up to 3 mL. The tissues were placed in cold saline to remove adhering blood and blotted on ash-free filter paper, and individual tissue weights were recorded. Relative organ weight was calculated as given below.

$$\begin{array}{c} \mathrm{Relative}\mathrm{organ}\mathrm{weight}\\ =\frac{\mathrm{Absolute}\mathrm{organ}\mathrm{weight}\left(g\right)}{\mathrm{Body}\mathrm{weight}\mathrm{of}\mathrm{mice}\mathrm{on}\mathrm{the}\mathrm{day}\mathrm{of}\mathrm{sacrifice}\left(g\right)}\times 100 \end{array}$$

The bacterial densities in the tissues were determined by homogenization in 0.9% sterile NaCl, serially diluted, and plated on MacConkey agar. For preparing histological slides, tissue sections were cut and fixed in 10% formalin solution. Histological slides were prepared by hematoxylin and eosin (H&E) staining procedures as described by Nath et al. (2017). The slides were examined under a light microscope EVOS1XL imaging system.

### Degradation of Pyrene in *in vitro* Conditions With Succinate as Co-substrate

*Klebsiella pneumoniae* AWD5 was tested for pyrene degradation in Bushnell Haas (BH) broth. 1 mL of cell suspension (O.D_600_ = 0.5, CFU/mL = 5.5 × 10^7^), which was maintained in Luria–Bertani (LB) broth, was inoculated into 0.005% pyrene-spiked BH broth. Also, the effect of succinate was determined in BH broth with 0.1% succinate amended with 0.005% pyrene and inoculated with bacterial culture. The flasks were incubated at 130 rpm in the dark at 30°C for 21 days. The biodegradation efficiency of pyrene was measured after every 7 days. Pyrene was extracted with ethyl acetate, which was added to the medium at a ratio of 1:2 (v/v), and the supernatant was extracted after incubation of 18 h and evaporated in the dark and resuspended in 1 mL of ethyl acetate. Quantitative analysis of pyrene degradation was analyzed using GC-MS.

### Pot Experiment (Soil Sampling and Pyrene Extraction)

The uncontaminated soil was collected from a field site in Dorgakona, South Assam, India, and 1 kg of dried soil was filled in ethanol-sterilized pots as described previously (Kotoky and Pandey, 2020; Singha and Pandey, 2020). *T. erecta* L. seeds were surface sterilized using 2% sodium hypochlorite (NaOCl) for 2–3 mins with subsequent washing in distilled water. Pyrene (98% purity, SIGMA-ALDRICH) at a concentration of 200 mg/kg was dissolved in ethyl acetate and spiked in the soils put in the pot. It was mixed thoroughly, air-dried, and kept to allow the solvent to evaporate. Three seedlings 15 days old were planted per pot to four different sets, (i) plant (control), (ii) plant + AWD5, (iii) plant + pyrene, and (iv) plant + pyrene + AWD5, where cell suspensions of *K. pneumoniae* AWD5 (5 mL, O.D_600_ = 0.6, CFU/mL = 5.5 × 10^7^) were applied to the soil. The experiments were carried out in triplicate in ambient greenhouse conditions and irrigated equally to make up the water loss. The experiments were terminated after observation for 60 days and the roots and shoots of the plants were separated and washed subsequently with distilled water, and growth parameters were measured and dried in an oven to measure the dry weight. The roots were separated and dried overnight in an oven to measure the dry root weight (DRW). Nitrogen (N) content in soil was assessed using standard procedures (Jackson, 1973), phosphorus (P) content was estimated by the ammonium molybdate method (Murphy and Riley, 1962), carbon (C) content was assessed as described by Navarro et al. (1993), and potassium (K) content was determined by the flame photometric method (Jankowski and Freiser, 1961). Pyrene was extracted at the end of the experiment from pots. 10 g of soil sample was suspended in 25 mL of ethyl acetate and thoroughly mixed and vortexed for nearly 1 h and incubated in a rotary shaker for 18 h. Then, the supernatant was filtered and collected and air-dried in the dark. Rhizospheric soils of all four different sets that were tightly adhered to the roots were separated and taken for DNA extraction for metagenomic sequencing.

### Analytical Method for Analysis of Pyrene Degradation

The residual pyrene percentage was determined by gas chromatography–mass spectrometry (GC-MS). GC-MS analysis was performed using a Clarus 680 gas chromatograph with the Clarus 600 C mass spectrometer system. The column used was Elite-5MS Capillary Column (length 60 m, I. D: 0.25 mm) with a phase reference (5% diphenyl polysilphenylene siloxane). Helium (99.99%) was the carrier gas with a flow rate and injection volume of 1 mL/min. The temperature of the column was held at 70°C for 5 min and increased at a rate of 4°C/min to 350°C and held for 10 mins. The mass spectrometer was operated in ionization mode (EI) at 70 electron volts (EV). The temperature of the injector was 270°C, and the detector was a sealed long-life photomultiplier.

The pyrene degradation rate was calculated as follows:

$$\mathrm{Degradation}\left(\%\right)=\frac{\begin{array}{c} \mathrm{Initial}\mathrm{concentration}\mathrm{of}\mathrm{pyrene}\\ -\mathrm{Final}\mathrm{concentration}\mathrm{of}\mathrm{pyrene} \end{array}}{\begin{array}{c} \mathrm{Initial}\mathrm{concentration}\mathrm{of}\mathrm{pyrene} \end{array}}\times 100$$

### Effect of AWD5 Treatment and Rhizoremediation on the Bacterial Community of *T. erecta* L.

Metagenomic DNA was isolated from 250 mg rhizospheric soil samples of each different set using a commercially available kit (NucleoSpin Soil). DNA samples (2 μL) were resolved on Agarose gel (0.8%) at 120 V for ∼60 min, and 1 μL of the sample was loaded for determining the A260/280 ratio in NanoDrop. Nextra XT Kit (Illumina Inc.) was used for the amplicon libraries, which tracks as per 16S metagenomic sequencing library preparation protocol. 16S rDNA gene-specific for the bacterial V3–V4 regions was amplified using primers F (5′-GCCTACGGGNGGCWGCAG-3′) and R (5′-ACTACHVGGGTATCTAATCC-3′). The libraries were sequenced using a 2 × 300-bp Miseq library. Amplification was done targeting 16S rDNA V3–V4 region-specific bacteria. 3 μL of PCR product was resolved on 1.2% agarose gel at 120 V for approximately 60 min or till the samples reached 3/4th of the gel. The amplicon that has passed quality check (QC) with the Illumina adaptors was amplified using primers (i5 and i7) that increase the multiplexing index sequence along with common adapters that are required for cluster generation (P5 and P7) according to standard Illumina chemistry. Further, amplicon libraries were purified by AMPureXP beads and quantified using a Qubit fluorometer. The amplified libraries were analyzed for quantity and quality check on the 4200 Tape Station system (Agilent Technologies) using a D1000 Screen tape. The libraries obtained from the main peaks were loaded on MiSeq at 10–20 pM concentration for cluster generation and sequencing. Template fragments to be sequenced in both forward and reverse directions were sequenced by paired-end sequencing on Miseq. Binding of samples to complementary adapter oligos was done using kit reagents on the paired-end flow cell. The paired-end sequences were analyzed using Quantitative Insights into Microbial Ecology (QIIME) (Caporaso et al., 2010) to provide taxonomic abundance. Sequences from all the samples clustered into Operational Taxonomic Units (OTUs) using Uclust at 97% sequence similarity, and each cluster represents a Species. The taxonomies were aligned to OTUs by aligning reads using Greengenes Database (McDonald et al., 2012). According to the evidence of taxonomic assignments, coverage, and GC content, the assembly was further grouped into bacterial taxa up to species level.

### Bioinformatics and Ecological Analysis of Microbial Communities

Ecological and statistical analyses were analyzed using QIIME (Caporaso et al., 2010), PAST (Hammer et al., 2001), and R packages RAM (version 1.2.1.3). Canonical correspondence analysis (CCA) was performed using the R package vegan (version 2.4-2). CCA was used to examine the influence of plant growth attributes including DRW, nitrogen (N), and phosphorus (P) on the structure of bacterial communities. For the CCA plot analyses, the correlation of the canonical axes with the explanatory matrix was determined with the general permutation test [vegan:permutest (); nperm = 999; default conditions]. Alpha diversity indices (Shannon diversity, Simpson diversity, Evenness, and Chao-1 richness estimation) were estimated using QIIME pipeline and PAST3, calculated based on the OTU table deriving from Illumina sequencing. Alpha diversity (Shannon–Wiener index) is generally used to measure bacterial diversity (Shannon and Weaver, 1949). It was calculated based on the OTU data of the whole bacterial community at the genus level, and a rarefaction curve was generated. Also, beta-diversity analysis was estimated by computing weighted UniFrac and unweighted UniFrac distances between the samples to generate principal coordinate analyses (PCoA). Also, global beta-diversity was analyzed using PAST3 to analyze the abundance difference of each OTU between different treatments.

### Accession Numbers

Metagenomic sequences had been deposited in NCBI, at Sequence Read Archive (SRA) under the accession number SRP55701; Bioproject ID: PRJNA480361; sample name: A_CONTROL; SRA: SRS3606947; Biosample: SAMN09635316; sample name: BAWD; SRA: SRS3606948; Biosample: SAMN09635317; sample name: CPyr; SRA: SRS3606950; Biosample: SAMN09635318; sample name: AWDPyr; SRA: SRS3606949; Biosample: SAMN09635319.

## Results

### Test for Non-pathogenicity of *K. pneumoniae* AWD5

**FIGURE 1:**
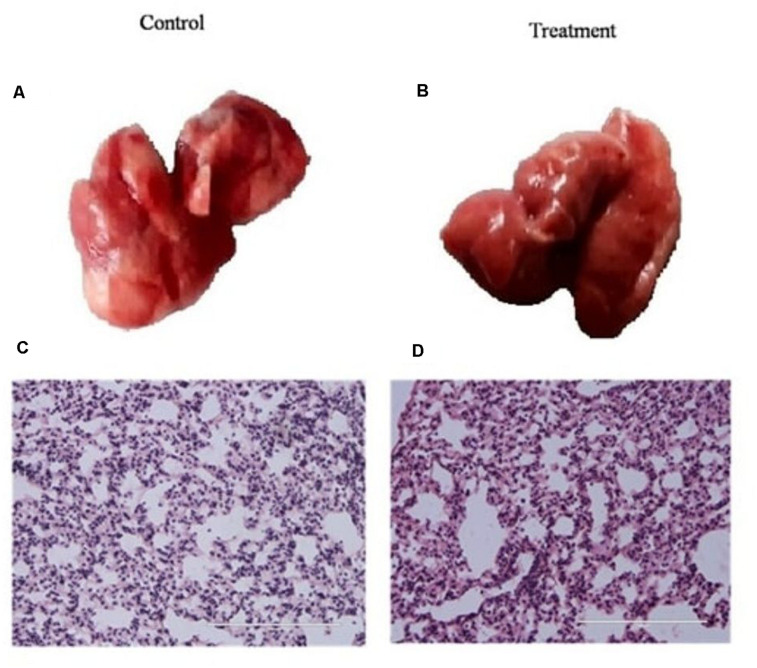
Histology of lung tissue in **(A)** control mice, **(B)** *K. pneumoniae* AWD5 treated mice, and **(C,D)** alteration in the tracheal index of both groups of mice. Scale bar = 100 μm. Histological images are representative of independent experiments. Data represents mean ± SD of three independent experiments. Number of mice *n* = 6, 3 controls, and 3 treated mice, respectively.

Before using *K. pneumoniae* AWD5 for pot trials, the virulence of the isolate was tested using a mouse model. The mice were infected with *K. pneumoniae* AWD5 and weighed daily until mice were sacrificed. The mice challenged with AWD5 (5.5 × 10^7^) survived the infection when the experiment was terminated, and also there was no bacteremia. In histological examinations, the lung parenchyma was clear, lacy, and essentially devoid of inflammatory cells in both control and treated mice, with no significant difference observed in the tracheal index between the two groups (Figure 1). Blood, spleen, and liver were also found to be sterile after AWD5 treatment as the organ homogenates were never detected with viable AWD5 cells during the study. The body weights of both control mice and *K. pneumoniae* AWD5-treated mice remained almost the same throughout the experiment. Also, the relative organ weight of the liver was almost similar as compared with the control mice (Supplementary Table 1).

### Analysis of Pyrene Degradation With Succinate as Co-substrate

**FIGURE 2:**
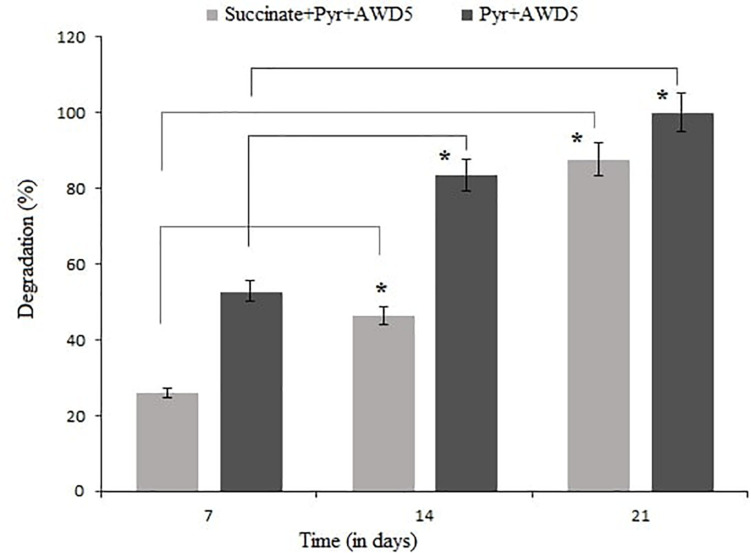
Degradation of pyrene by *K. pneumoniae* AWD5 after 21 days, with succinate as carbon source (*p < 0.05).

The degradative ability of *K. pneumoniae* AWD5 was analyzed in BH broth spiked with pyrene and succinate amended with pyrene, and the content was harvested thrice. The rate of degradation was observed to be 52.81% after 7 days and 83.48% after 14 days of incubation, and pyrene was completely removed after 21 days of incubation in pyrene-spiked medium. Different degrees of degradation ranging from 25.88 to 87.5% were observed in the minimal medium after 21 days of incubation in the presence of succinate (Figure 2). Pyrene utilization by AWD5 was confirmed by its removal from pyrene-amended BH broth with a corresponding increase in bacterial growth.

### Effect of *K. pneumoniae* AWD5 Inoculation on *T. erecta* L. Growth and Soil Properties

**FIGURE 3:**
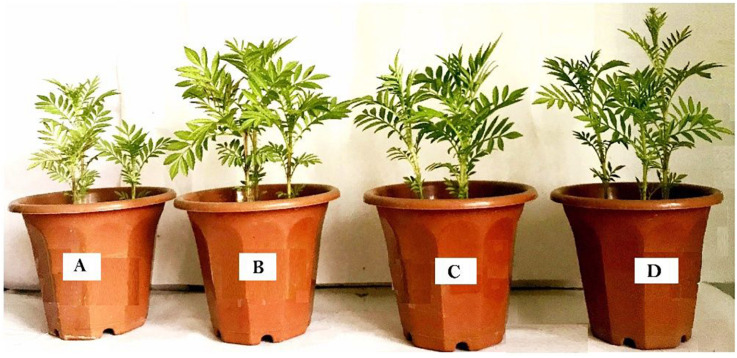
Effect of *K. pneumoniae* AWD5 on *T. erecta* L. during 60 days. **(A)** Control soil, **(B)** AWD5-inoculated soil, **(C)** Pyrene-amended soil, **(D)** AWD5 inoculation + pyrene-amended soil.

The growth of *T. erecta* L. plants was observed by inoculating AWD5 in pyrene-amended and unamended soil. Inoculation of *K. pneumoniae* AWD5 resulted in 14.64, 80.56, 3.26, and 45.35% increases in root length, dry root weight, shoot length, and dry shoot weight of *T. erecta* L., respectively, as compared with control. There was no significant difference in the plant growth parameters between the treatments having pyrene and AWD5 + Pyr. However, there was a 14.42% decrease in fresh shoot weight in pyrene-amended soil as compared to control, while inoculation of AWD5 in pyrene-amended soil improved plant growth by 7.96% as compared with control. Also, the moisture content of the plant was decreased by 10.32% due to pyrene stress; however, the presence of AWD5 in pyrene-amended soil increased by 43.87% as compared with control (Figure 3 and Table 1).

**TABLE 1:** Growth parameters of *T. erecta* L. plant with different treatments.

Parameters	Control	AWD5	Pyrene	AWD5 + Pyr
Shoot length (cm) ^†^	19.61 ± 4.83	21.25 ± 1.30*	19 ± 4.01	19.86 ± 5.14
Fresh shoot weight (g) ^†^	1.96 ± 0.43	2.93 ± 0.53 *	1.72 ± 0.36	2.57 ± 0.41*
Dry shoot weight (g) ^†^	0.86 ± 0.40	1.25 ± 0.11 *	0.76 ± 0.23	0.81 ± 0.13
Root length (cm) ^†^	17.24 ± 0.12	19.58 ± 1.76*	17.08 ± 1.76	18.5 ± 0.71*
Fresh root weight (g) ^†^	0.80 ± 0.18	1.59 ± 0.33*	0.85 ± 0.12	0.95 ± 0.11
Dry root weight (g) ^†^	0.36 ± 0.04	0.65 ± 0.16*	0.40 ± 0.16	0.54 ± 0.04*
Plant moisture content (g) ^†^	1.55 ± 0.84	2.34 ± 0.46*	1.39 ± 0.30	2.23 ± 0.37*
pH	6.6 ± 0.28	6.6 ± 0.28	6.65 ± 0.21	6.45 ± 0.11
Organic carbon (kg/ha)	0.345 ± 0.12	0.45 ± 0.028*	0.435 ± 0.176*	0.445 ± 0.021*
N (kg/ha)	109.76 ± 22.72	156.8 ± 2.83*	106.8 ± 17.98	109.76 ± 22.17
P (kg/ha)	120 ± 8.33	138 ± 5.05*	128 ± 6.87*	131 ± 10.12*
K (kg/ha)	148.5 ± 3.53	161.5 ± 3.54*	150.5 ± 4.94	156 ± 4.24*

Each value represents mean ± standard deviation of three replicates, *P ≤ 0.05. ^†^Average values given as for per plant.

The ability of ornamental plant *T. erecta* L. to remove pyrene from contaminated soils was examined. Inoculation of AWD5 enhanced the availability of organic carbon (C), nitrogen (N), phosphorus (P), and potassium (K) in soil. It relatively increased C (0.45 kg/ha), N (156.8 kg/ha), P (138 kg/ha), and K (161.5 kg/ha) contents of the soil and therefore stimulated plant growth. It showed a better growth rate, though pyrene contamination affected soil N content and had no influence on soil pH. The pH values ranged from 6.45 to 6.65. The bioavailability of pyrene (200 mg/kg) did not vary with either soil pH. Total C, P, and K contents of the soil were enhanced with bacterial inoculation and amendment of pyrene as compared with control.

### Analysis of Pyrene Degradation in Soil

**FIGURE 4:**
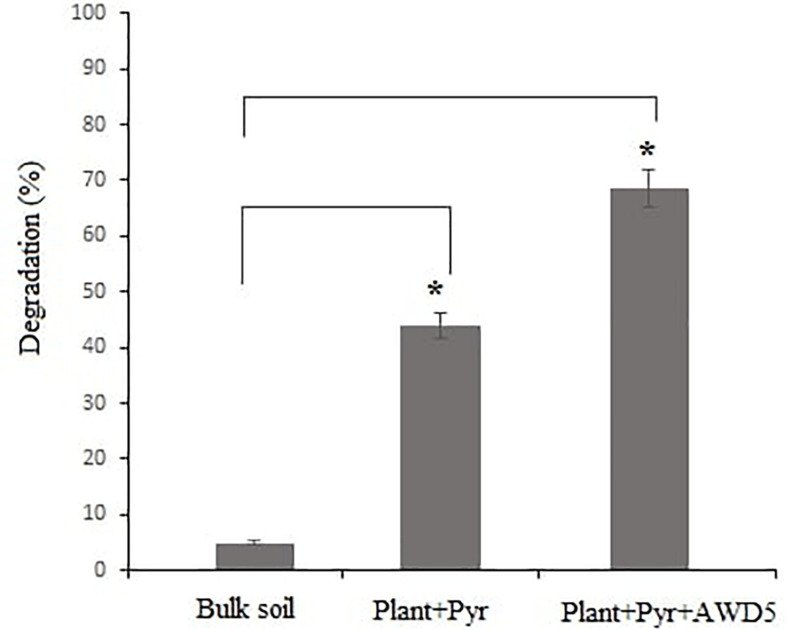
Degradation percentage of pyrene (after 60 days) in different treatments (*p < 0.05).

After 60 days of the experiment, the treatments including control soil (no plant) were analyzed for the quantification of pyrene removal in soil. In the presence of *T. erecta* L., the pyrene removal was observed to be 43.99% whereas inoculation of AWD5 led to an increase in percent degradation which was recorded as 68.61%, while in unplanted control soil removal of pyrene was recorded as 4.19% (Figure 4). In GC-MS analysis, the chromatogram showed the peak (retention time—39.44) for pyrene which decreased in the test samples after 60 days of incubation (Supplementary Figure 1).

### Taxonomic Analysis of the Microbial Community of *T. erecta* L. During Pyrene Degradation

**FIGURE 5:**
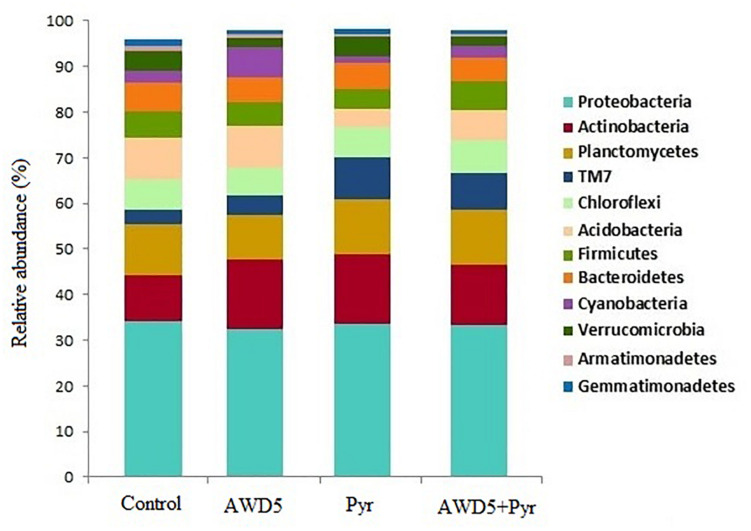
Comparative analysis between the rhizospheric soil (Control), *K. pneumoniae* AWD5 (AWD5), pyrene-amended soil (Pyr), and *K. pneumoniae* AWD5 + pyrene (AWD5 + Pyr)-inoculated rhizospheric soil of *T. erecta* L. plant at the phylum level taking a 0.5% threshold.

The taxonomic analysis of the rhizosphere microbial community revealed the presence of 37 bacterial phyla across all four samples. The most abundant phyla were found to be Proteobacteria followed by Actinobacteria, Planctomycetes, TM7, Chloroflexi, Acidobacteria, Firmicutes, Bacteroidetes, Cyanobacteria, Verrucomicrobia, Armatimonadetes, Gemmatimonadetes, OD1, and many other bacterial phyla with less than 1% abundance. The relative abundance at phylum-level distribution across samples for these 13 taxa is presented in Figure 5. The relative abundance of Verrucomicrobia was observed to be reduced by treatment of *K. pneumoniae* AWD5 (2.04%) and AWD5 + Pyr (2.00%), as compared with control (4.14%) and Pyr (4.50%). Relative abundance of phyla varied under different treatments, including Acidobacteria (control 9.28%; AWD5 9.29%; Pyr 4.02%; AWD5 + Pyr; 6.66%), and Verrucomicrobia (control 4.14%; AWD5 2.04%; Pyr, 4.50%; AWD5 + Pyr, 2.00%).

At the class level, Alphaproteobacteria belonging to phylum Proteobacteria was most abundant in all soil samples followed by Actinobacteria. The bacterial classes identified in soil samples are given in Supplementary Figure 2. The relative abundance of *Sphingobacteria* was found to be higher in pyrene-amended soil (Pyr, 0.87%; AWD5 + Pyr, 0.60%), as compared with control (0.42%) and AWD5 (0.42%). Gammaproteobacteria was found to be increased with AWD5 treatment as compared with control and reduced with pyrene amendment.

**FIGURE 6:**
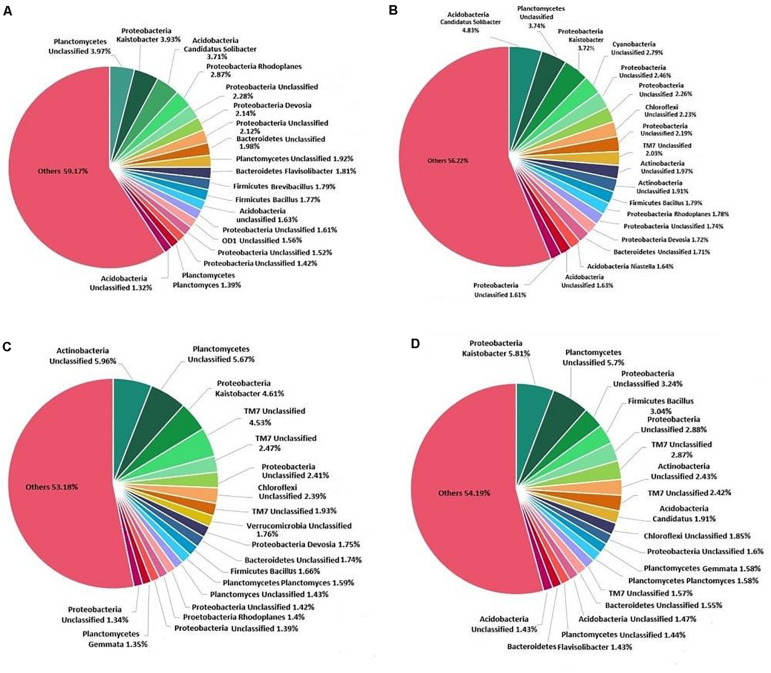
Composition and relative abundance of major bacterial taxa at the genus level: **(A)** control soil, **(B)** *K. pneumoniae* AWD5-inoculated soil, **(C)** pyrene-amended soil, and **(D)** *K. pneumoniae* AWD5 + pyrene-treated soil.

At the genus level, *Kaistobacter* was found to be most abundant followed by an unclassified genus of order WD2101 belonging to phylum Planctomycetes (Figure 6). Treatment of *K. pneumoniae* AWD5 in soil increased the relative abundance of *Bacillus* (AWD5, 1.80%; AWD5 + Pyr, 3.05%) as compared with control (1.77%) and Pyr (1.66%). The relative abundance of an unclassified genus belonging to phylum Actinobacteria was found to be increased with AWD5 treatment (AWD5, 0.93%; AWD5 + Pyr, 1.07%) as compared with control (0.63%) and Pyr (0.56%). Also, the relative abundance of *Ochrobactrum* increased with the treatment of AWD5, as in control it was only 0.001%, while in AWD5 treatment and in AWD5 + Pyr treatment, it was 0.113% and 0.123%, respectively. The genus was altogether absent in pyrene (without AWD5)-amended soil. *Candidatus solibacter* of phylum Acidobacteria was observed to be dominant in AWD5-treated soil (AWD5, 4.82%), and its abundance was reduced in pyrene treatment (Pyr, 0.99%; AWD5 + Pyr, 1.91%) as compared with control (3.71%). Furthermore, some of the genera identified as *Pelotomaculum*, *Chryseobacterium*, *Methanomassiliicoccus*, and unclassified genera of phyla belonging to Actinobacteria and Chloroflexi were observed to be present in AWD5-treated soils (AWD5 and AWD5 + Pyr), while these were not detected in the control and pyrene-amended soil. The relative abundance of *Kaistobacter* was improved in AWD5 + Pyr-treated soil (AWD5 + Pyr, 5.79%) as compared with control (3.92%), AWD5 treatment (3.71%), and pyrene-amended soil (Pyr, 4.60%). Pyrene-amended soil was dominated by an unclassified genus from phylum Actinobacteria (Pyr, 5.95%), and relative abundance of the genus was found to be reduced (control, 1.05%; AWD5, 1.96%; AWD5 + Pyr, 2.42%). Besides, treatment with pyrene-amended soil increased the relative abundance of an unclassified genus of phylum TM7 (Pyr, 4.52%) as compared with control (1.18%), AWD5 (2.02%), and AWD5 + Pyr (2.41%). The abundance of *Aeromicrobium* of phylum Actinobacteria was enhanced with a pyrene amendment (Pyr, 0.927%), as compared with control (0.064%), AWD5 (0.011%), and AWD5 + Pyr (0.004%). An unclassified genus of order Gammaproteobacteria was also observed to be increased in pyrene-amended soil (Pyr, 0.021%) as compared with control (0.013%), AWD5 (0.007%), and AWD5 + Pyr (0.005%).

### Comparative Analysis of the Rhizobacterial Community Structure

**FIGURE 7:**
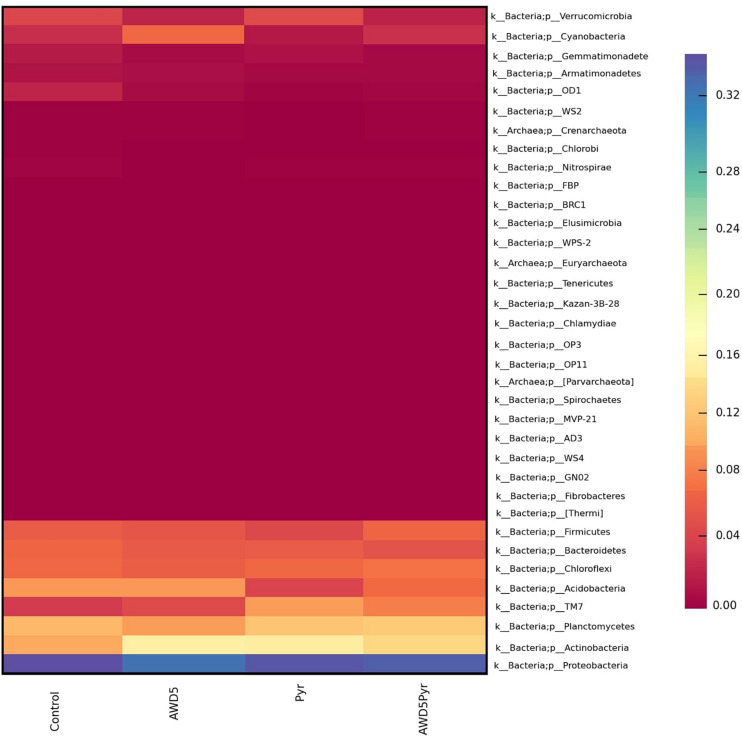
Heat map showing the relative abundance of various phyla in samples of rhizospheric soil of *T. erecta* L. (control), AWD5 (soil with *K. pneumoniae* AWD5), Pyr (soil amended with pyrene), and AWD5 + Pyr (pyrene-amended soil + *K. pneumoniae* AWD5).

The alpha diversity of each test soil was estimated with Shannon–Wiener (H), Simpson (1-D), Chao-1, and evenness indices. These indices were interpreted at the genus level, which assesses the diversity and richness of the species of each soil, and these indicated relative abundances and species richness present for the samples. The highest diversities based on the Shannon–Wiener index obtained in soils were control soil (*H* = 4.78), *K. pneumoniae* AWD5 (*H* = 4.643), pyrene-amended soil (*H* = 4.602), and AWD5 + Pyr (*H* = 4. 581). A lower Simpson diversity index was obtained in pyrene-amended soil (1-D = 0.9813) while Chao-1 richness in this same sample was 435.8. Alpha rarefaction curves showed a similar profile for all the samples, which were plotted with the average number of OTUs at each interval against the size of the (Table 2) samples. The curve gives the richness of the microbial species in all the samples. The variation in bacterial diversity was significant (*p* ≤ 0.05) between different treatments as analyzed using Tukey’s multiple-comparison test, where most of the diversity indices were found to be significantly different among treatments (Supplementary Table 2). The control sample and AWD5-treated sample (AWD5) were plotted in the upper part of the graph (Supplementary Figure 3). Global beta diversity was estimated at the genus level to illustrate sample diversity across different soil samples. PAST3 analysis showed Whittaker (0.065015), Harrison (0.021672), Cody (74), Routledge (0.01886), Wilson–Shmida (0.18328), and Mourelle (0.061094) values. The PCoA-based weighted and unweighted UniFrac distance matrix was used to estimate community-level diversity between the soil samples. Weighted UniFrac displays the phylogenetic distance of a relative abundance of dominant OTUs of the samples and showed that samples were segregated distantly in one co-ordinate indicating the presence of common microorganisms (Supplementary Figure 4). The variance defined by the first, second, and third components in weighted UniFrac distance was 50.37, 34.36, and 15.26%, respectively. The unweighted PCoA revealed a relatively less variation along the first three axes. The variance elucidated was 44.37, 28.7, and 26.93% for the first, second, and third components, respectively. Control, pyrene, and AWD5 + Pyr samples were grouped in one co-ordinate while the AWD5-inoculated sample formed a separate cluster in the PC3 cluster, which suggests that a unique microbial diversity exists as compared to other soil samples (Supplementary Figure 5). A heat map was created to illustrate the relative abundance of OTUs at the phylum level, which signifies dissimilarity between the microbial communities (Figure 7).

**TABLE 2:** Diversity indices of the rhizospheric bacterial community with different sets of treatment.

Alpha diversity	Taxa_S	Dominance_D	Simpson_1-D	Shannon_H	Evenness_e^H/S	Chao-1
Control	430	0.01356	0.9864	4.78	0.2771	439
AWD5	363*	0.01529*	0.9847	4.643*	0.2862	389.1*
Pyr	422	0.01868*	0.9813	4.602*	0.2363	435.8*
AWD5 + Pyr	400*	0.01773*	0.9823	4.581*	0.244	431.9*

*Indicates significance at p ≤ 0.05.

**FIGURE 8:**
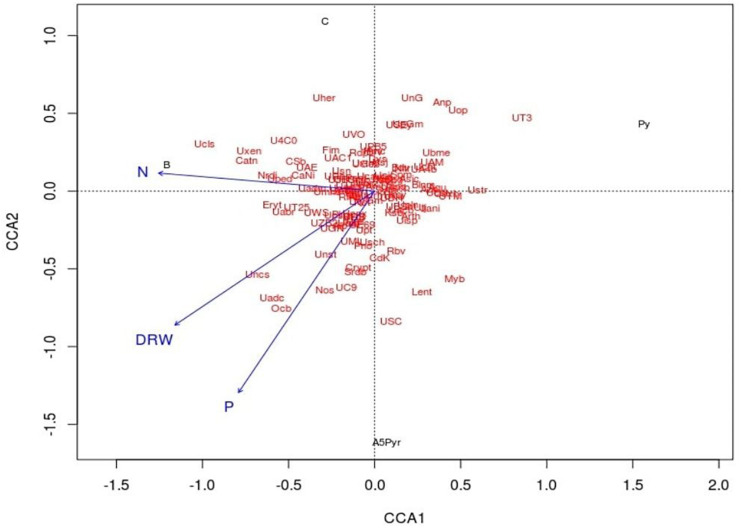
Canonical correspondence analysis ordination plot of the relationship between soil bacterial community structure and environmental variables (DRW indicates root dry weight, N indicates nitrogen, and P indicates phosphorus). Solid circle represents soil samples (Cont indicates control soil, AWD5 indicates soil inoculated with *K. pneumoniae* AWD5, Pyr indicates pyrene-amended soil, and AWD5 + Pyr indicates *K. pneumoniae* AWD5 + pyrene-amended soil).

Canonical correspondence analysis (CCA) was used to assess the relative influence of the microbial community structure on selected environmental variables DRW, N, and P. In the CCA biplot, the arrow signifies the direction and magnitude of variables. Here, the growth attributes were represented by an arrow, and the length of the arrow signifies the extent of the variable’s variance. All four samples were distributed on two ordinate axes. The plant growth attributes such as P, N, and DRW content in the soil samples were placed in the same coordination CCA2 connected with the treatment of AWD5. The direction of an arrow shows the direction of an increase in corresponding plant growth attributes. The relative abundance of some phyla Actinobacteria, Acidobacteria, Crenarchaeota, Cyanobacteria, Proteobacteria, Verrucomicrobia, and TM7 had a greater impact on the presence of AWD5 and also correlated with nitrogen content as demonstrated by the arrow in the CCA plot. Furthermore, DRW was related to the abundance of Cyanobacteria and influence Acidobacteria and Proteobacteria abundance. P content enriched Acidobacteria, Actinobacteria, Cyanobacteria, Proteobacteria, and Chloroflexi. However, the pattern of bacterial communities’ structure has a negative correlation with pyrene treatment, and Pyr and AWD5 + Pyr were segregated in different coordinates of CCA1 (Figure 8).

### Correlation of Bacterial Abundances With Bacterial Inoculation and Pyrene Degradation

**FIGURE 9:**
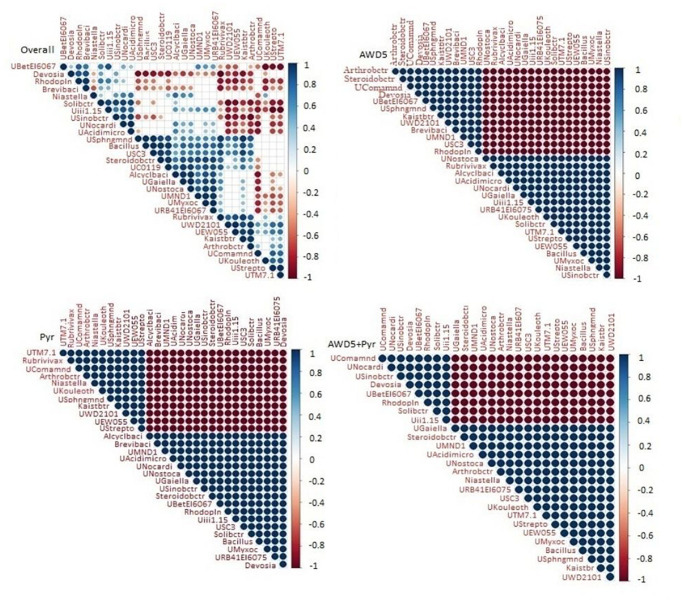
Spearman’s rank correlation between microbial communities of four different soil 460 samples. Blue nodes signify positive correlation and red signifies negative correlation.

The microbial abundances were correlated at the genus level among individual bacterial species in the different soil samples using Spearman’s rank correlation (Figure 9). The correlation analysis of all four samples showed a positive correlation between genus *Kaistobacter* and *Arthrobacter*, *Steroidobacter* with unclassified Chloroflexi (UC0119), and unclassified Myxococcales (UMyxoc) irrespective of the presence of AWD5 and pyrene. *Bacillus* showed positive co-occurrence with unclassified Acidomicrobiia (UAcidimicro), unclassified Nostocaceae (UNostoca), and unclassified Myxococcales of phylum Proteobacteria (UMyxoc). The abundance of *Devosia* showed a negative correlation with unclassified Sphingomonadaceae (USphngmnd), unclassified Chloroflexi (UC0119), *Rubrivivax*, and *Kaistobacter*.

Correlation analysis of control with AWD5 inoculation showed a positive correlation of *Kaistobacter* with *Arthrobacter* and *Steroidobacter*. The relative abundance of *Bacillus* was also shown to interrelate with unclassified Acidomicrobiia (UAcidimicro) of phylum Actinobacteria and unclassified Nostocaceae (UNostoca) of phylum Cyanobacteria. The presence of Chloroflexi in AWD5-treated soil was shown to be positively related to unclassified Nostocaceae and unclassified Acidomicrobiia. In the presence of AWD5, *Candidatus solibacter* showed a negative correlation to Arthrobacter, Steroidobacter, unclassified Sphingomonadaceae, *Kaistobacter*, unclassified Betaproteobacteria_MND1, and unclassified TM7. Further, *Devosia* showed a negative correlation with unclassified Sphingomonadaceae (USphngmnd), unclassified Chloroflexi (UC0119), Rubrivivax, Kaistobacter (Kaistbtr), and Candidatus Solibacter (Solibctr) with unclassified Phycisphaerae (UWD2101). Pyrene-amended soils were found to enhance the abundance of Actinobacteria, and hence *Arthrobacter* was found to have a positive correlation with *Niastella*, unclassified Sphingomonadaceae (USphngmnd), *Kaistobacter*, unclassified Phycisphaerae WD2101 (UWD2101), and unclassified EW055 (UEW055) of phylum TM7. Another unclassified genus Acidomicrobiia (UAcidimicro) of phylum Actinobacteria was positively related to the presence of unclassified Nostocaceae (UNostoca) of phylum Cyanobacteria, *Bacillus*, unclassified Myxococcales (UMyxoc) of Proteobacteria, and unclassified Gaiellales (UGaiella) of Actinobacteria. The abundance of phylum Actinobacteria was positively correlated with TM7, Bacteroidetes, Proteobacteria, and others.

## Discussion

In this study, the effects of non-pathogenic *K. pneumoniae* AWD5 on the degradation of pyrene were determined, when applied through *T. erecta* L. Also, its effect was assessed on plant-growth and rhizosphere bacterial community structure to evaluate it as a potential candidate for PAH rhizoremediation. The non-pathogenic nature of *K. pneumoniae* AWD5 was confirmed in the mouse model before employing in greenhouse applications. The experiment confirmed that the strain was incapable of causing lung infection to infected animals. The architecture of the lungs did not show pathogenic alteration that may indicate pathogenic infection. This lack of animal pathogenicity of environmental isolates of *Klebsiella* spp. had been observed previously also, as in *Klebsiella* sp. strain Kd70 which showed incapability to cause urinary tract infection in Kd70-infected mice (Dantur et al., 2018). While environmental isolate *K. pneumoniae* 342 was demonstrated as pathogenic, it was observed to be less virulent than the typical *K. pneumoniae* clinical isolate (Fouts et al., 2008).

*Klebsiella pneumoniae* AWD5 was observed to degrade pyrene efficiently in the minimal medium even in the presence of succinate, which signifies that the presence of other substrates does not hinder pyrene degradation although it extended the duration of degradation. Hence, pyrene can be metabolized by *K. pneumoniae* AWD5 despite the presence of other co-substrates, such as succinate which is found in root exudates and is present as an alternate C source for rhizospheric bacteria. Kotoky and Pandey (2020) suggested that the presence of other carbon sources like succinate could stimulate the growth of bacterial isolate and enhance BaP degradation. Additional carbon sources like succinate enhanced degradation of phenanthrene by nearly 90% in 24 h by *Massilia* sp. WF1 (Wang et al., 2016). HMW-PAH degradation was enhanced by *Cellulosimicrobium cellulans* CWS2 when succinate was supplied as co-substrate as such LMW organic acids are the intermediate of various carbon sources (Qin et al., 2018). Succinate was observed to enhance the bacterial strain’s mobility in PAH degradation when it was used as a growth substrate (Grimm and Harwood, 1997). Furthermore, the sequestering soil matrix was shown to be dislocated in the presence of low-molecular-weight organic acids; therefore, the availability of PAHs in the soil might be influenced and enhance desorption (Ling et al., 2009).

The AWD5 bacterial strain enhanced plant growth in pyrene-amended soil and stimulated N content. Pyrene was rapidly degraded in pyrene-amended soils including bulk control soil; this may be due to abiotic loss of PAHs such as sequestration and volatilization, as suggested earlier by Meng and Zhu (2011). Phytodegradation has been a favorable strategy for the decontamination of PAHs in polluted soils (Denys et al., 2006). Different plant species like ryegrass and alfalfa had different promoting effects on PAH removal (Lee, 2013). *Medicago sativa*, *Brassica napus*, and *Lolium perenne* had shown to dissipate pyrene in soils by 32, 30, and 28%, respectively (D’Orazio et al., 2013). In the present work, *T. erecta* L. is being reported as a suitable plant for PAH bioremediation, and its potential for commercial use should be explored. In pot experiments, *T. erecta* L. plants appeared to grow normally in pyrene-amended soil; however, the root length, fresh shoot weight, and moisture content of the plants were observed to be slightly lower in comparison with control. *K. pneumoniae* AWD5 inoculation enhanced the plant biomass and moisture content of the plant. Pyrene along with bacterial inoculation (AWD5 + Pyr) enhanced the plant biomass as compared with pyrene-amended soil. There was no observable difference between the *T. erecta* L. plants irrespective of whether the soil was inoculated with bacteria and pyrene. Plants showed no significant difference in their growth in the presence or absence of pyrene, which indicated that the plants could tolerate pyrene at a concentration of 200 mg/kg of soil and showed good growth. Shoot length, root length, and plant biomass were observed to be promoted in the presence of AWD5 as the stress effects of pyrene were reduced as compared to control. In soil, pyrene had been reported to show an inhibitive effect on plant biomass. Alfalfa shoot biomass was observed to be 34% of the control with an increase in pyrene concentration (493 mg/kg) in the soil after 60 days of growth. Root biomass was also found to be reduced with a higher concentration of pyrene from 49 to 493 mg/kg soil (Fan et al., 2008). The root-and-shoot biomass of the *Sorghum bicolor* plant is increased by 14.5 and 21.27%, respectively, at 150 mg/kg pyrene concentration as compared with the control and reduced with a higher concentration of 300 mg/kg pyrene. *Sorghum bicolor* in combination with *Pseudomonas* bacterial strain has been reported to enhance the efficiency of pyrene removal rate (Rostami et al., 2017). The plant biomass may depend on the concentration of pollutants in soil. Accumulation of plant biomass could be affected by increasing the concentration of PAHs during plantation especially after 120–150 days of cultivation (Wang et al., 2014). Fire Phoenix plants during its growth period lessened the toxic effects of PAHs and reached its plant height with a minimum value after 120 days. The plant was found to be tolerant of PAHs, and maximum root and shoot inhibition effects were observed after 60 days with HMW-PAHs. The degradation rate of pyrene and chrysene reached 96.27 and 88.59%, respectively, after 60 days (Dai et al., 2020). Plant roots had been reported to change the soil chemical structure and enhanced microbial activities, which led to an effect of more reduction of PAHs (Xu et al., 2006).

At the end of the 60-day experiment, concentrations of pyrene in all sets were decreased. The removal of pyrene was higher in rhizospheric soil than in non-rhizospheric soil. Pyrene removal was found to be 43.99% by *T. erecta* L. plant alone, and the inoculation of bacterial strain AWD5 increased up to 68.61%. These results showed that an increase in pyrene degradation in soil may be attributed to *T. erecta* L. rhizosphere biodegradation, and inoculation of bacterial strain enhanced the removal of pyrene in the presence of *T. erecta* L. The pyrene removal percentage was found to vary from 69 to 85% after 60 days in the alfalfa rhizosphere while non-rhizosphere soil recorded 59% to 80%, which showed that the presence of roots stimulated the rate of degradation (Fan et al., 2008). It had been reported that ornamental plant *T. erecta* L. could be used for phytoremediation of PAHs and it had strong tolerance to HMW-PAHs and showed a remediation rate of 79.2–92.4% in 92 days in benzo(a)pyrene (BaP)-contaminated soils (Sun et al., 2011). *T. erecta* L. has branched root systems that offer large root surfaces for the growth of the population of microorganisms and extended biodegradation in the presence of plants. The rate of removal of BaP was higher in rhizospheric soil at different stages (seedling, flowering, and mature stage) of *T. erecta* L. growth and recorded as 2.7–26.8% (Sun and Zhou, 2016). Addition of corn plant and *Pseudomonas putida* MUB1 in treatment removed pyrene more than 80% in 20 days, which could have achieved this much level within 30 and 60 days if plant alone or bacterial alone was inoculated (Chouychai et al., 2009). Singha and Pandey (2017) demonstrated that *Pseudomonas aeruginosa* PDB1 stimulated the growth of *Jatropha curcas* in pyrene stress. Phytoremediation of PAH-contaminated sites with a specific group of bacteria earns more attention. Tall fescue root biomass was increased with inoculation of *Klebsiella* sp. D5A, though no significant difference in shoot biomass was observed. Concentrations of PAHs (C16–C21) were found to be reduced with *Klebsiella* sp. D5A-treated soil (Liu et al., 2016). *K. pneumoniae* strain PL1 isolated from soil was found to degrade 63.4% of 20 mg/L pyrene and 55.8% of 10 mg/L BaP in 10 days, respectively (Ping et al., 2014).

Since soil microbes play an important role in regulating ecosystem functions, and different plant species impact associated soil microbial communities (Sun et al., 2018), the changes in the microbial community structure of the *T. erecta* L. rhizosphere is being reported for the first time in this study, though it had been reported to be used in phytoremediation of BaP (Sun et al., 2011).

In AWD5-inoculated and pyrene-amended treatments, more than 19 bacterial phyla were present of which Proteobacteria was found to be dominant. Proteobacteria have been reported to be the dominant phylum in several environments, including soil, seawater, and petroleum hydrocarbon-contaminated sites (Zhang et al., 2010; Mukherjee et al., 2014). Bacteria belonging to phylum Proteobacteria were shown to be dominant in the bacterial community from estuarine sediment, which also possesses genes for pyrene degradation (Zhang et al., 2019). Abbasian et al. (2016) observed the abundance of Proteobacteria (32.1%) as dominating phyla followed by Actinobacteria (12.3%) in crude oil field soil. Actinobacteria was found to be the second dominant phylum in all the treatments. Actinobacteria was observed to be abundant in PAH-contaminated soil, which had been reported to be involved in the metabolism of hydrocarbons (Abbasian et al., 2016; Zhao et al., 2018). This phylum was reported to adapt to environmental stress, and it was found to be dominant in soils with < 10% organic matter, hydrocarbon, and diesel-polluted soils (Bell et al., 2013a; Bourceret et al., 2015). Actinobacteria are HMW-PAH degraders in soil, and 12–18% of Actinobacteria were reported in pyrene-contaminated soil, which showed their involvement in degradation (Deng et al., 2016). AWD5 inoculation enhanced the population of Acidobacteria, Cyanobacteria, and Actinobacteria as compared with control, while the pyrene amendment reduced the relative abundance of these bacterial groups. AWD5-inoculated treatment in the presence of pyrene further improved their relative abundance. Metagenomic analysis of Ba(P)-contaminated soil revealed a shift in the microbial community in favor of degradation of the xenobiotic compound when supplemented with bacterial consortium, as suggested previously (Kotoky and Pandey, 2020). The relative abundance of Firmicutes was observed to be increased in the AWD5-treated and pyrene-amended sample (6.38%) as compared with the pyrene-amended sample (4.34%). Actinobacteria, Proteobacteria, and Firmicutes had been reported to increase during PAH and petroleum hydrocarbon degradation (Fuentes et al., 2014). The relative abundance of Acidobacteria was enhanced with AWD5 treatment (9.29%) as compared with other treatments. Bioaugmentation with an aromatic hydrocarbon degrader and biostimulation with corn stover were reported to induce a shift in the native microbial community in favor of specific members of degraders like Bacteroidetes, Firmicutes, and Actinobacteria (Zafra et al., 2016). The abundance of phylum TM7 with AWD5 treatment showed 4.59% as compared with control (3.41%); higher abundance was marked in pyrene-amended soil (Pyr, 9.47%); and pyrene along with AWD5 treatment (AWD5 + Pyr) showed 7.92%. Phyla TM7 showed a higher abundance in pyrene-amended soil. Members of TM7 had been identified in bioremediating BTEX contaminated soils (Xie et al., 2011). Microbial composition of petroleum hydrocarbon-contaminated Chernozem soil showed a higher proportion of *Candidate*_*division*_TM7 (Liao et al., 2015).

Alphaproteobacteria exhibited the dominant bacterial class in all the treatments; however, pyrene-amended soil has higher relative abundance as compared with control and bacterial inoculation. This bacterial class was found to be the most abundant taxon with HMW-PAH-contaminated soils (Kuppusamy et al., 2016). An average of 20–50% of members of Alphaproteobacteria constituted the bacterial community in soils contaminated with aged PAHs (Zhang et al., 2011). Members of Alphaproteobacteria have been reported to play an important role in the network function in PAH-contaminated soils (Kaur et al., 2015). The abundance of Gammaproteobacteria was enriched with bacterial inoculation in the current study; the existence of this bacterial class has indicated degradation of PAHs in soil (Niepceron et al., 2013). On the other hand, an abundance of Betaproteobacteria when treated with AWD5 in pyrene-amended soil showed 5.77%, which revealed a higher abundance in pyrene-amended soil (Pyr, 6.44%) as compared with control (5.79%). The presence of Betaproteobacteria was reported to be suggestive of the completion of the degradation of PAHs (Lors et al., 2010). It has been reported that the abundance of Betaproteobacteria was found to be increased in an agricultural soil incubated for 90 days with phenanthrene, pyrene, or a mixture of seven PAHs, which is indicative of their degradation capacity (Niepceron et al., 2013). Alpha-, Beta-, and Gammaproteobacteria have been reported to utilize aliphatic and aromatic compounds and enhance their abundance during biodegradation of soils contaminated with petroleum hydrocarbons (Auti et al., 2019). Hou et al. (2015) detected an increase in the abundance of Gammaproteobacteria with inoculation of *Klebsiella* sp. D5A. The relative abundance of *Sphingobacteria* was observed to be enhanced with a pyrene amendment in soil. Ventorino et al. (2018) reported an increase in the abundance of *Sphingobacteria* in hydrocarbon contaminated soils than non-contaminated soils. Moreover, members of *Sphingobacteria* were recovered from polluted soil and found to be involved in aromatic and aliphatic hydrocarbon degradation (Kaur et al., 2015; Abbasian et al., 2016).

At the genus level, the abundance of *Kaistobacter* was found higher in pyrene treatment (pyrene-amended soil, 4.60%); also, bacterial inoculation enhanced its abundance (AWD5 + Pyr, 5.79%). *Kaistobacter* was found abundant as indigenous microbes contaminated with antimony-rich tailings dump (Xiao et al., 2016). The addition of AWD5 enhanced the abundance of *Bacillus* (AWD5, 1.80%; AWD5 + Pyr, 3.05%). This may be because the inoculation of AWD5 could modulate the rhizospheric community and increased the abundance in favor of xenobiotic compound degradation. A similar observation has been recently reported for PAH degradation (Kotoky and Pandey, 2020). *Bacillus* had been identified as metabolically versatile and degrades aromatic compounds (Cebron et al., 2011). It has been reported to remediate phenanthrene, and the presence of root exudates made it more competitive during degradation (Haichar et al., 2008). The relative abundance of *Verrucomicrobia* was observed to be higher in pyrene-amended soil (4.50%), while its abundance was lower in other treatments. The abundance of *Verrucomicrobia* was observed higher in hydrocarbon-contaminated soil, indicating the importance of the phylum in the bioremediation of contaminated soils (Shahi et al., 2016; Ventorino et al., 2018). Microbial communities are an integral part of the plant rhizosphere and contribute to the growth of the plants as well as for bioremediation of pollutants (Kotoky et al., 2018). The study revealed that the growth and development of *T. erecta* L. and microbial community structure in the rhizosphere were not affected by AWD5 inoculation. Therefore, AWD5 has the potential to use as a degrader for contaminated soil that would enrich the rhizosphere community with degraders in the rhizosphere and promote plant growth in the rhizosphere.

The Shannon diversity index was used to estimate whether *K. pneumoniae* AWD5 inoculation and pyrene amendment in soils affect the bacterial richness and diversity. A higher Shannon index shows a more diversified bacterial community (Shannon and Weaver, 1949). The most diversified bacterial community soil (Shannon index) and highest species richness (Chao-1) were observed in the control soil. The *H*-value for control was highest, which showed better diversity of the bacterial community, in terms of both richness and abundances. Observed OTUs and Shannon diversity index were reduced in pyrene-amended soil as compared with the control treatment; this may be indicative of the presence of efficient bacterial species that can resist or degrade pyrene rapidly in the contaminated soil, thus leading to less bacterial diversity. Shannon and Simpson’s indices showed that soil incubated with a mixture of PAHs for a long duration changed the bacterial community and showed lower bacterial diversity (Muangchinda et al., 2018). The inverse Simpson diversity index was observed to reduce significantly in rhizoremediation of petroleum hydrocarbon-contaminated soil inoculated with *Klebsiella* sp. D5A and *Pseudomonas* sp. SB as compared with the control soil (Hou et al., 2015). Bell et al. (2013b) described that the decrease in bacterial diversity could not slow down the degradation rate of hydrocarbons and diversity is less essential for actual hydrocarbon degradation. The Shannon diversity index of consortium growing with naphthalene was found to be lower, which reflected that different microorganisms were involved to adapt to the changes of the environment with different PAHs (Garrido-Sanz et al., 2019). The Shannon diversity index was further increased with AWD5 treatment in pyrene-amended soil, which may indicate that the strain was capable of pyrene degradation, thereby enriching the bacterial community. Changes in microbial diversity with inoculation of AWD5 may be associated with a higher abundance of pyrene degrading bacteria in the treated soil.

The weighted UniFrac matrix indicated the presence of a common microbial community in the samples. PCoA-weighted UniFrac analysis showed the community structure of a consortium SWO, isolated from crude oil-contaminated seawater different from control seawater, which could degrade a mixture of PAHs including HMW-PAHs (Muangchinda et al., 2018). The unweighted UniFrac metric showed the *K. pneumoniae* AWD5-treated *T. erecta* L. rhizosphere sample cluster separately, which is suggestive of different rhizobacterial community structures due to abundance of particular species in the soil while other samples were clustered together. The unweighted UniFrac distance analyzed the phylogenetic distance between the samples without considering their relative abundance (Gauchotte-Lindsay et al., 2019).

Analysis of possible linkage between plant growth attributes and microbial community structure by CCA plot analysis showed that *K. pneumoniae* AWD5-treated soil was correlated with the available nitrogen content and influenced the abundance of bacterial community structure. The addition of a bacterial community, Mycobacteria, has been reported to increase bacterial biodiversity in the soil and enhanced the efficiency of pyrene degradation whereas *Pseudomonas* or *Sphingomonas* treatment improved the efficiency of naphthalene and phenanthrene degradation (Hennessee and Li, 2016). Microbial diversity changes with pyrene-amended soil and N, P, and DRW have a negative correlation with the pyrene contaminant. The soil microbial community has been found to vary with oil contamination levels, and CCA showed a negative correlation of phosphorus and oil contamination and positive relation of nitrogen content with pristine soils (Liang et al., 2009). Earlier, the sediment microbial communities were observed to be positively correlated with PAHs, phosphorus, and nitrogen content, and PAHs were identified as the main factor that shaped the microbial community structure (Yan et al., 2019).

To further elucidate the correlation between bacterial communities’ structures, most abundant OTUs based on relative abundance across all samples were analyzed by Spearman correlation analysis. The relative abundance of dominant member *Kaistobacter* belonging to Alphaproteobacteria in AWD5-treated soil has a positive correlation with different bacterial species like *Arthrobacter* of class Actinobacteria and *Brevibacillus* of Firmicutes. *Devosia* of Alphaproteobacteria exhibited a negative correlation with unclassified Sinobacteraceae of class Gammaproteobacteria in pyrene-amended samples. Betaproteobacteria and Bacteroidetes were reported to be enriched in red soil at different concentrations of pyrene (Ren et al., 2015). Phylum Proteobacteria and Bacteroidetes abundances were significantly correlated with PAH concentration in the sediment (Yan et al., 2019). *Devosia* of Alphaproteobacteria has a positive relation with *Rhodoplanes* belonging to Alphaproteobacteria in AWD5-treated samples. *Rubrivivax* of Betaproteobacteria exhibited a positive relation with *Kaistobacter* of Alphaproteobacteria in pyrene-amended samples. Previously, correlation analysis has suggested that Alphaproteobacteria shows the highest positive relation, while Betaproteobacteria and Gammaproteobacteria show an inverse correlation with pyrene concentration (Vinas et al., 2005). In all the treatments, the relative abundance of *Candidatus Solibacter* has a strong correlation with unclassified Sinobactereraceae, unclassified Nocardioidaceae, and unclassified Acidimicrobiales. Also, *Bacillus* has a strong correlation with *Steroidobacter*, *Kaistobacter*, *Rubrivivax*, and *Alicyclobacillus*. The presence of specific microbial communities could indicate specific habitats and forecast microbial response for the effective degradation of PAHs. AWD5 thus modulated the composition of bacterial communities during the *T. erecta* L. rhizosphere-assisted bioremediation of pyrene.

## Conclusion

Inoculation of *K. pneumoniae* AWD5 in pyrene-amended soil and plant–microbe interaction in the rhizosphere of *T. erecta* L. exhibited efficient rhizoremediation of pyrene. This indicated that the isolate is suitable for rhizoremediation and capable of degrading pyrene even in the presence of succinate, present as co-substrate. Inoculation of *K. pneumoniae* AWD5 enhanced the growth of the plants and maintained the soil microbial community. Application of metagenomics techniques illustrated toward understanding the indigenous microbial community in pyrene-contaminated soil. Based on metagenome data analysis, the soils with different treatments were observed to be dominated by Proteobacteria, followed by Actinobacteria, Planctomycetes, and others with relatively low numbers. Bacterial inoculation was observed to restore the microbial population. Also, the presence of pyrene might have played a key role in conditioning the soil community and ultimately led to an increase in the relative abundance of bacterial taxa which contribute to the degradation of PAHs. Also, it is important to experimentally observe the pathogenicity of bacterial strains before application in agricultural practices. Thus, this bacterial strain and its association with *T. erecta* L. might help in developing an efficient strategy to improve bioremediation approaches. This result provided the perceptions in understanding the ecology of bioremediation of PAH-exposed soil.

## Data Availability Statement

The datasets presented in this study can be found in online repositories. The names of the repository/repositories and accession number(s) can be found below: https://www.ncbi.nlm.nih.gov/genbank/, SRP55701; Bioproject ID: PRJNA480361; Sample name: A_CONTROL; SRA: SRS3606947; Biosample: SAMN09635316; Sample name: BAWD; SRA: SRS3606948; Biosample: SAMN09635317; Sample name: CPyr; SRA: SRS3606950; Biosample: SAMN09635318; Sample name: AWDPyr; SRA: SRS3606949; Biosample: SAMN09635319.

## Ethics Statement

The animal study was reviewed and approved by Animal Ethics Committee, Assam University, Silchar, India.

## Author Contributions

JR has performed the experiments, data curation, and analysis and wrote the manuscript. PP has conceptualized the work, supervision, and writing and editing of the manuscript. YC supervised the experiment on mice. KB assisted in mouse experiment and data generation. JR and YC contributed equally to this work. All authors contributed to the article and approved the submitted version.

## Conflict of Interest

The authors declare that the research was conducted in the absence of any commercial or financial relationships that could be construed as a potential conflict of interest.

## Supplementary Material

Supplementary Table 1 Relative organ weight of mice.

Supplementary Table 2 *p*-Values of Alpha diversity indices calculated using the Tukey’s Multiple comparison test (*p* ≤ 0.05).

Supplementary Figure 1 Gas chromatography–mass spectrometry chromatogram of degradation of pyrene in (A) control bulk soil, (B) *T. erecta* L. planted soil after 60 days of incubation and (C) *T. erecta* L. planted soil inoculated with *K. pneumoniae* AWD5.

Supplementary Figure 2 Relative abundance of major taxonomy at class level of rhizospheric soil (Control), *K. pneumoniae* AWD5 (AWD5), pyrene amended soil (Pyr) and *K. pneumoniae* AWD5 + pyrene (AWD5 + Pyr) inoculated rhizospheric soil of *T. erecta* L. plant.

Supplementary Figure 3 The alpha rarefaction curve for the samples- Control, AWD5, Pyr, AWD5 + Pyr. Each curve illustrates the cumulative OTU number at a phylogenetic distance of 0.03.

Supplementary Figure 4 A principal coordinates plot based on weighted Unifrac distances of all samples.

Supplementary Figure 5 A principal coordinates plot based on unweighted Unifrac distances of all samples.
