# Supplementary material for: Rhizodegradation of Pyrene by a Non-pathogenic Klebsiella pneumoniae Isolate Applied With Tagetes erecta L. and Changes in the Rhizobacterial Community
Source: Front Microbiol. 2021 Feb 23;12:593023. doi: 10.3389/fmicb.2021.593023 (PMC7940843; doi:10.3389/fmicb.2021.593023)
Supplement: Supplementary Table 1 — Relative organ weight of mice. [file Table_1.DOCX]

**Supplementary data**

**Table S1**. Relative organ weight of mice

| **Set** |  | **Relative organ weight g/kg (body weight) x 100±SEM** |  |  |
| --- | --- | --- | --- | --- |
|  | **Final body weight (g)** | **Liver** | **Lung** | **Spleen** |
| Control | 31.17±.003 | 1.324±.121 | 5.689±.004 | 3.6833±.0003 |
| Treatment (*Klebsiella pneumonia* AWD5) | 31.13±.041 | 1.361±.004* | 5.404±.007* | 3.6146±.0002 |

Values are mean ± SEM of three independent experiments with three technical replicates each.

⁎ Indicates significant difference from respective controls at p < 0.05.

**Table S2:** p values of Alpha diversity indices calculated using the Tukey's Multiple comparison test (p≤0.05)

| **Alpha**  **diversity** | **Taxa_S** | **Dominance_D** | **Simpson_1-D** | | **Shannon_H** | **Evenness_e^H/S** | **Chao-1** |
| --- | --- | --- | --- | --- | --- | --- | --- |
| AWD5 | 0.04 | 0.05 | | 0.07 | 0.01 | 0.07 | 0.05 |
| Pyr | 0.06 | 0.04 | | 0.07 | 0.02 | 0.07 | 0.03 |
| AWD5+Pyr | 0.05 | 0.03 | | 0.05 | 0.02 | 0.08 | 0.03 |

Significance between the diversity parameters for different treatments is analyzed (Tukey’s multiple comparison test). Other than Evenness most of the diversity indices were found to be significantly different (p≤0.05) including Dominance and Shannon index, between the treatments.


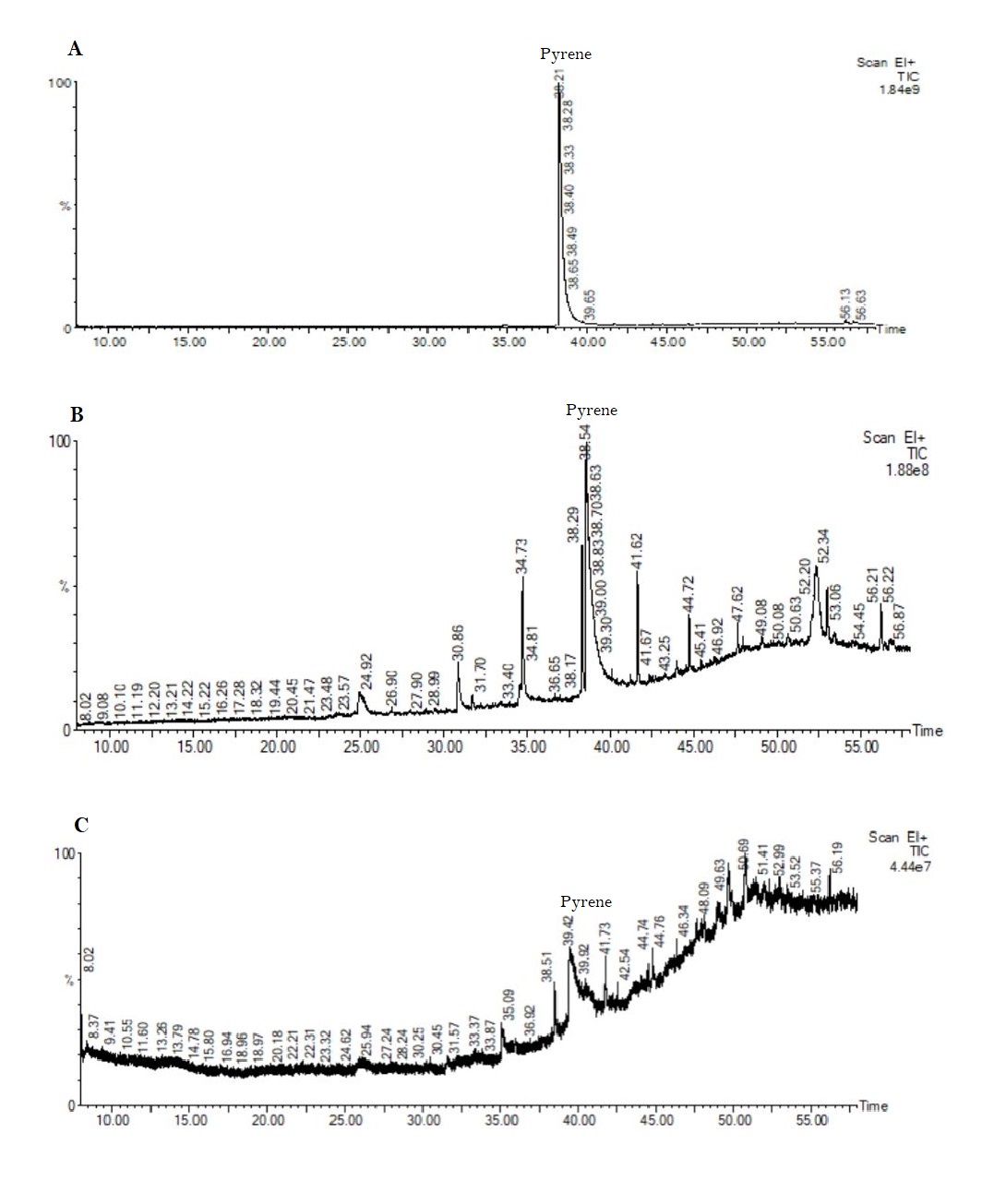


**FIGURE S1.** GC-MS chromatogram of degradation of pyrene in (A) control bulk soil, (B) *T. erecta* L. planted soil after 60 days of incubation and (C) *T. erecta* L. planted soil inoculated with *K. pneumoniae* AWD5.


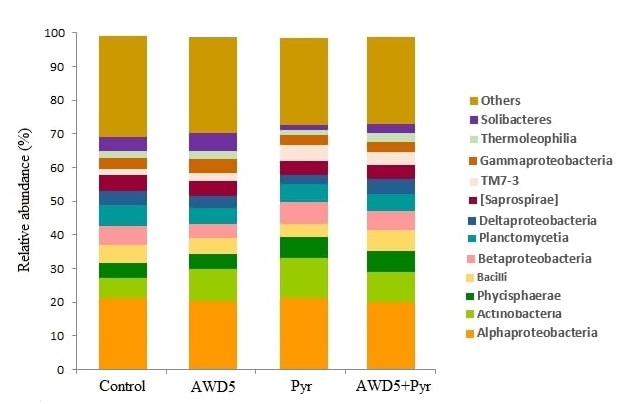


**Figure S2**: Relative abundance of major taxonomy at class level of rhizospheric soil (Control), *K. pneumoniae* AWD5 (AWD5), pyrene amended soil (Pyr) and *K. pneumoniae* AWD5 + pyrene (AWD5+Pyr) inoculated rhizospheric soil of *T. erecta* L. plant


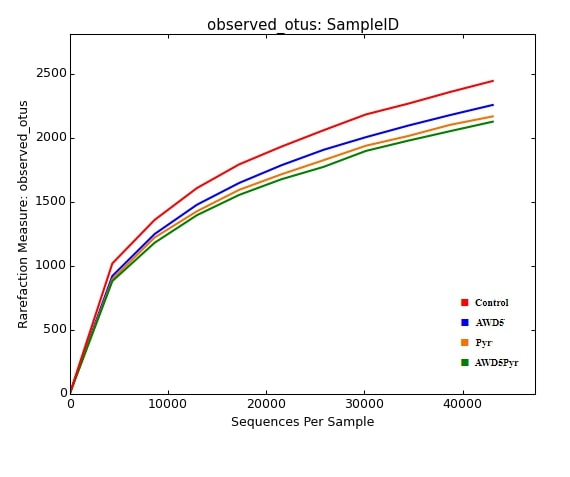


**FIGURE S3**: The alpha rarefaction curve for the samples- Control, AWD5, Pyr, AWD5+Pyr. Each curve illustrates the cumulative OTU number at a phylogenetic distance of 0.03


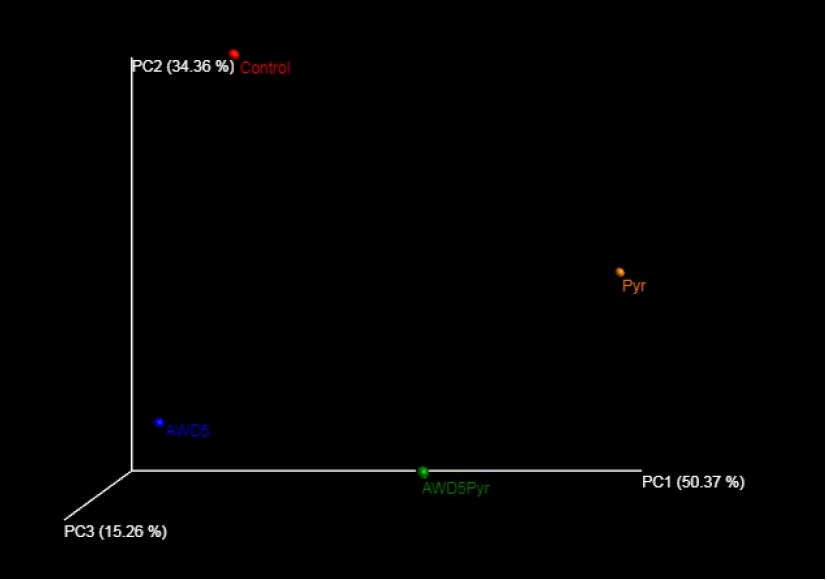


**FIGURE S4**: A principal coordinates plot based on weighted Unifrac distances of all samples


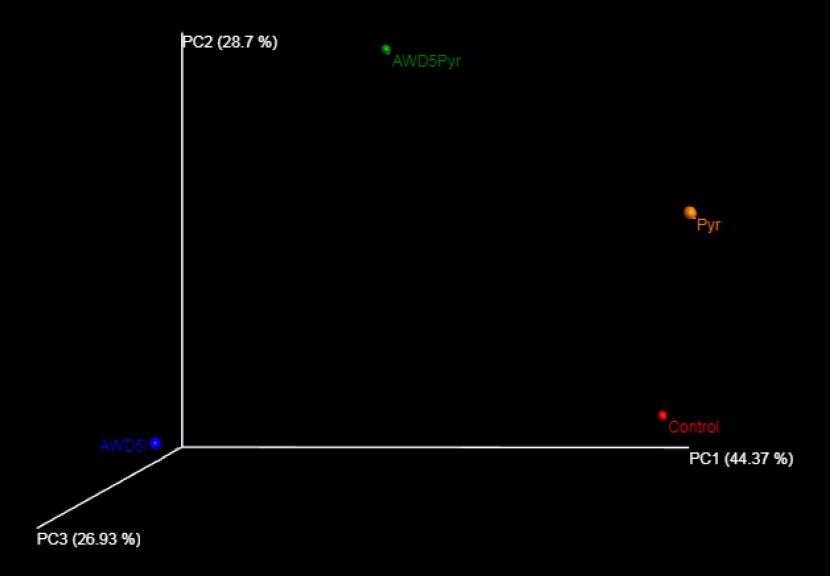


**FIGURE S5:** A principal coordinates plot based on unweighted Unifrac distances of all samples
